# GPCRdb in 2021: integrating GPCR sequence, structure and function

**DOI:** 10.1093/nar/gkaa1080

**Published:** 2020-12-03

**Authors:** Albert J Kooistra, Stefan Mordalski, Gáspár Pándy-Szekeres, Mauricio Esguerra, Alibek Mamyrbekov, Christian Munk, György M Keserű, David E Gloriam

**Affiliations:** Department of Drug Design and Pharmacology, University of Copenhagen, Universitetsparken 2, 2100 Copenhagen, Denmark; Department of Drug Design and Pharmacology, University of Copenhagen, Universitetsparken 2, 2100 Copenhagen, Denmark; Department of Drug Design and Pharmacology, University of Copenhagen, Universitetsparken 2, 2100 Copenhagen, Denmark; Medicinal Chemistry Research Group, Research Center for Natural Sciences, Budapest H-1117, Hungary; Department of Drug Design and Pharmacology, University of Copenhagen, Universitetsparken 2, 2100 Copenhagen, Denmark; Department of Drug Design and Pharmacology, University of Copenhagen, Universitetsparken 2, 2100 Copenhagen, Denmark; Department of Drug Design and Pharmacology, University of Copenhagen, Universitetsparken 2, 2100 Copenhagen, Denmark; Medicinal Chemistry Research Group, Research Center for Natural Sciences, Budapest H-1117, Hungary; Department of Drug Design and Pharmacology, University of Copenhagen, Universitetsparken 2, 2100 Copenhagen, Denmark

## Abstract

G protein-coupled receptors (GPCRs) form both the largest family of membrane proteins and drug targets, mediating the action of one-third of medicines. The GPCR database, GPCRdb serves >4 000 researchers every month and offers reference data, analysis of own or literature data, experiment design and dissemination of published datasets. Here, we describe new and updated GPCRdb resources with a particular focus on integration of sequence, structure and function. GPCRdb contains all human non-olfactory GPCRs (and >27 000 orthologs), G-proteins and arrestins. It includes over 2 000 drug and in-trial agents and nearly 200 000 ligands with activity and availability data. GPCRdb annotates all published GPCR structures (updated monthly), which are also offered in a refined version (with re-modeled missing/distorted regions and reverted mutations) and provides structure models of all human non-olfactory receptors in inactive, intermediate and active states. Mutagenesis data in the GPCRdb spans natural genetic variants, GPCR-G protein interfaces, ligand sites and thermostabilising mutations. A new sequence signature tool for identification of functional residue determinants has been added and two data driven tools to design ligand site mutations and constructs for structure determination have been updated extending their coverage of receptors and modifications. The GPCRdb is available at https://gpcrdb.org.

## INTRODUCTION

G protein-coupled receptors (GPCRs) account for 4% (799 (1) / 20 595 (2)) of human genes and mediate the actions of two-thirds (342/515) of hormones and neurotransmitters (3). Their abundant modulation of human physiology is mirrored in medicine where 34% of marketed drugs act on GPCRs (4). Hence, GPCRs are studied by very large basic receptor research and drug discovery communities. The GPCR database, GPCRdb currently serves >4 000 researchers every month with multi-disciplinary reference data, data visualisation/analysis, tools to design experiments and dissemination of datasets upon publication. GPCRdb is compliant with the FAIR principles (5), provides all its code as open source, gives full free access to all its resources and publishes open access whenever possible.

GPCR sequence, structure and function data is increasing rapidly from advances in e.g. cryo-electron microscopy (cryo-EM) (6), deep mutational scanning (7), genome sequencing (8) and signal protein profiling (9,10). The curation and integration of such large datasets require extensive work—typically beyond the capacity in the individual research group or study. In this article, we describe recent updates of GPCRdb infrastructure—section by section—that integrate and infer GPCR sequence, structure and function data at the receptor and residue levels. We hope that this will aid the community to reach a better understanding of receptor mechanisms and how they may be exploited for drug discovery.

## MATERIALS AND METHODS

### Structure annotation and sequence alignment

New GPCR structures are imported to a development database instance upon release in the Protein Data Bank (PDB) (11) along with draft data on e.g. ligands, auxiliary fusion and signalling proteins, and preferred chain. These data are manually inspected, and further annotations are added before (now monthly) release in GPCRdb to allow researchers to select relevant receptor structures. The first and last residue positions are annotated in each receptor segment—the transmembrane helices 1–7 (TM1–7), helix 8 (H8) and intra-/extra-cellular loops 1–2 (ICL/ECL1–2)—and used to define their length in the sequence alignments and homology models in GPCRdb. For all human GPCRs lacking a structure we infer each segment's start and end from receptors with either similar sequence motives/lengths or overall sequence. When a new structure is published for an already annotated receptor, the structural annotation is updated, if necessary. For example, the first structure with a native ICL3 sequence can correct the cytosolic ends of the flanking TM5 and TM6 previously affected by the use of a protein fusion facilitating crystallography (12). The sequence annotation of species orthologs is inferred from the human ortholog based on an automated sequence alignment.

### Generic residue numbering

The generic residue numbering uses a structural investigation to confirm or correct the receptor residue that corresponds to the reference position (designated the number 50) in each of TM1–7 using the sequence-based numbering scheme for the given GPCR class (A: Ballesteros-Weinstein (13), B1: Wootten (14), C: Pin (15), D1 (16) and F: Wang (17)) and in H8 and ICL/ECL1–2 (first defined by GPCRdb) (18,19). To ensure that the residue numbers are generic also when one of two compared receptor structures contains a helix bulge or constriction that cause an offset in a sequence-based numbering of the following residues, the observed structural single-position gap is represented by a corresponding gap in a structure-based sequence alignment (18). In contrast to sequence-based alignments, which align residues by their position in the gene product and therefore are suited for evolutionary studies, the unique alignment and generic numbering of residues that are equivalent in their structural position are designed for residue-structure-function correlation.

### Extending amino acid identity with property conservation in sequence alignments and signatures

Amino acid properties were defined based on polarity, helical propensity and size. Subsequently, we defined a distance for each property from the receptor backbone by counting the bonds from each residue's Cα atom to the sidechain atom with the given property (for multiple atoms with the same property we used the most terminal). Finally, residue property conservation was measured in percent for each of the in total 56 groups of or single amino acids that share a property and differ by none or one in their backbone distance. Furthermore, we implemented numeric amino acid descriptors—specifically frequently used ‘z-scales’ derived from principle component analysis of 10 experimental and 16 calculated properties (20). For each of the residue positions and five *z*-scales, the average and standard deviation value is calculated and the separation between the conserved and non-conserved residues is evaluated with a two-tailed *t*-test.

### Structure statistics

We implemented a ‘Structure model statistics’ page containing root-mean-square deviation (RMSD) values of the latest GPCRdb model (based on another receptor template) to the first experimental structure of the given receptor and state as well as a link to documentation and scripts used for the calculations. For this publication, we also downloaded models from the latest release (2019-12-20) of the RosettaGPCR’s Github page (https://github.com/benderb1/rosettagpcr) and database site (http://www.meilerlab.org/index.php/gpcrmodeldb) which have identical models. Thirty seven receptors had attained their first experimental inactive state structures since the launch of the GPCRdb homology modelling pipeline and could therefore be evaluated. However, 16 models could not be used for validation, as RosettaGPCR uses alignment.fasta files (from https://github.com/benderb1/rosettagpcr) that include the target structure among its templates. For the remaining 21 GPCRdb and corresponding RosettaGPCR models, RMSD values were calculated to the experimental structure for the following receptor segments and atoms: 7TM all atoms, 7TM backbone atoms, ICL1, ICL2, ECL1, ECL2, ECL3 and H8 (Supplementary Spreadsheet 1). To ensure a comparable measure, only atoms present in the experimental target structure and in both models were included in the RMSD calculation. The superposition was based on the 7TM backbone atoms defined by GPCRdb sequence alignments.

### Ligand database update

All GPCR ligands and their biological activities were imported from ChEMBL version 27 (21) using the ChEMBL web services (22) which were queried using ChEMBL identifiers. These were obtained from the UniProt (2) accession codes for all receptor entries in GPCRdb. To filter out ligand activities for which the principal receptor target is ambiguous, the ‘target type’ was restricted to ‘SINGLE PROTEIN’. ChEMBL entries were filtered to contain a ‘standard_value’, a ‘pchembl_value’, no comments regarding the data validity, and no ‘inconclusive’ tag in the activity comments (see https://www.ebi.ac.uk/chembl/faq). Ligand physicochemical properties and commercial availability data were retrieved from the PubChem database, as described previously (23).

## OVERVIEW OF DATA TYPES AND SIZES

**Figure 1.**
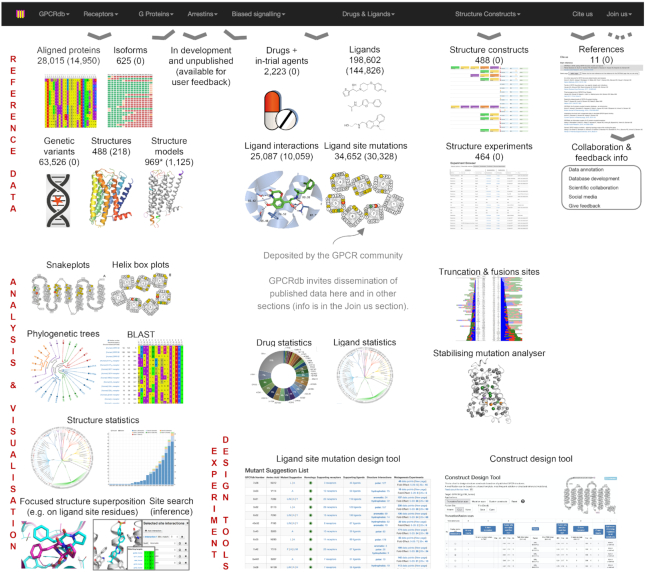
Overview of GPCRdb resources. For data (top), the numbers listed first are the current number of entries and those in parenthesis are for the previous GPCRdb update published in *Nucleic Acids Research* (23). A range of analysis and visualization tools (mid) allow for generation of new results and figures for publication or presentation. Data driven tools (bottom mid-right) can help to design new experiments. *The number of structure models decreases, and the number of refined structures increases as more receptors and their states (inactive, intermediate and active) are covered by experimental structures (see the Structures and Structure models sections).

Figure 1 shows an overview of the expansion of data types and sizes in GPCRdb since the last update published in January 2018 in *Nucleic Acids Research* (23). GPCRdb now includes all 398 non-olfactory GPCRs, 16 G-proteins and four arrestins in human and >28 000 species orthologs. For receptors, GPCRdb also stores 625 distinct isoforms, which can have diverse signalling and drug responses (24), and 63 526 natural genetic variants (25). The database holds 2223 drugs or in-trial agents (4) and 198 602 ligands. The structural data spans 488 GPCR structures, 969 GPCR structure models, 25 087 GPCR-ligand interactions and 488 structure constructs and experiments. The *in vitro* mutagenesis data spans mutations/chimera of GPCR-G protein interfaces (26), 34 652 ligand site mutations and thermostabilising mutations (12). Furthermore, GPCRdb has data driven tools to identify sequence signatures of functional residue determinants (see below), to mutate ligand binding sites (27) and to design constructs and experiments for structure determination (12). Finally, GPCRdb offers a range of analysis tools (see below and the GPCRdb web site). The reference for each data and tool is described in a new GPCRdb section Cite Us (see below and Table 1). Below, all new and updated resources are described in the same order as in the GPCRdb web site sections and subsections.

**Table 1.** References used to cite different GPCRdb resources

Scope	Title	Authors	Year	Journal	Ref
Main reference (combine with reference for the below)	GPCRdb in 2021: Integrating GPCR sequence, structure and function	Kooistra *et al.*	2021	*Nucleic Acids Research*	(36)
Drugs, drug targets and indications	Trends in GPCR drug discovery: new agents, targets and indications	Hauser AS *et al.*	2017	*Nature Reviews Drug Discovery*	(4)
G protein alignments and GPCR-G protein interface	Selectivity determinants of GPCR-G-protein binding	Flock T *et al.*	2017	*Nature*	(26)
Generic residue numbering	Generic GPCR residue numbers - aligning topology maps while minding the gaps	Isberg V *et al.*	2015	*Trends in Pharmacological Sciences*	(18)
Genetic variants	Pharmacogenomics of GPCR Drug Targets	Hauser AS *et al.*	2018	*Cell*	(25)
GPCR structure models and ligand statistics	GPCRdb in 2018: adding GPCR structure models and ligands	Pandy-Szekeres G *et al.*	2018	*Nucleic Acids Research*	(23)
Isoforms (from the same GPCR gene)	Combinatorial expression of GPCR isoforms affects signalling and drug responses	Marti-Solano *et al.*	2020	*Nature*	(24)
Ligand site mutations and structural interactions	Integrating structural and mutagenesis data to elucidate GPCR ligand binding	Munk C *et al.*	2016	*Current Opinion in Pharmacology*	(27)
Sequence alignments, receptor similarity, structure superposition and site search tools	GPCRdb: an information system for G protein-coupled receptors	Isberg V *et al.*	2016	*Nucleic Acids Research*	(33)
Structure constructs and experiments	An online resource for GPCR structure determination and analysis	Munk C *et al.*	2019	*Nature Methods*	(12)
Tools and good practices for mutagenesis experiments, structural template selection, receptor similarity and ligand interaction analyses	GPCRdb: the G protein-coupled receptor database - an introduction	Munk C *et al.*	2016	*British Journal of Pharmacology*	(34)

## RECEPTORS

### Receptor selection table combining classification with function and data availability

Receptor selection has hitherto been supported through searches by name or browsing of a hierarchical classification of GPCR classes, ligand type and receptor families sharing endogenous ligand. Here, all GPCRdb pages including receptor selection have been updated with a new receptor table allowing for a data driven selection that combines classification with function, such as G protein coupling, and availability of e.g. structural templates and ligands. Receptor lists (UniProt identifiers) can be saved and imported allowing analyses across GPCRdb pages and over time.

### Sequence alignments and residue property conservation

GPCRdb has structure-based sequence alignments of the GPCR transmembrane helices 1–7 (TM1–7), helix 8 (H8) and intra-/extra-cellular loops 1–2 (ICL/ECL1–2), which lengths and reference positions for generic residue numbering are defined from a manual annotation of crystal/cryo-EM structures (see Materials and Methods and (18)). For all human GPCRs lacking a structure, the segments length and residue numbering is curated based on receptors with similar sequence motifs or overall sequence, and species orthologs are aligned by inferring the information from the human reference protein. The continuous curation by experts – incorporating also user feedback – and coverage of the non-olfactory GPCRome in several species provide a GPCR community reference for many methods and studies that involve sequence analysis.

The amino acids conservation, shown under a GPCRdb sequence alignment, have herein been complemented with conservation and consensus of residue properties and mean values of numeric amino acid descriptors, ‘z-scales’ (20) (Materials and Methods). Each residue property (polarity, helical propensity and size) is further subgrouped to differ by none or only one in their backbone distance for the most characteristic or terminal sidechain atom. For example, charged residues are divided into 12 groups/singletons, three for any charge (with any, 3–4 and 4–5 bond distances), three for negative charge (with any, 3 and 4 bond distances) and six for positive charge (with any, 4, 4–5, 5, 5–6, 6 bond distances). These amino acid groups for residue property conservation, like ligand-based pharmacophores and many previous amino acid descriptors (28), were designed to correlate common sequence features with the ability to mediate a shared molecular interaction and function, such as the binding of a ligand or G protein or intra-receptor contacts stabilising a conformational state determining activity or signalling bias.

### Generic residue number tables

Generic residue numbers allow different receptors to be compared at the residue-level by assigning a common index for the receptor segment (TM1–7, H8 or ICL/ECL1–2) and a position therein that is equivalent in sequence or, in the case of GPCRdb numbering, in structure (see Materials and Methods and (18)). GPCRdb's generic residue tables can include any GPCRs and the numbering format spans the receptor-specific number in the canonical UniProt sequence isoform, the sequence-based TM1–7 residue numbering scheme for the given GPCR class (A: Ballesteros-Weinstein (13), B1: Wootten (14), C: Pin (15), D1 (16) and F: Wang (17)) and the structure-based GPCRdb scheme which also includes H8 and structurally conserved positions in ICL/ECL1–2 (19) (added in this update). A generic residue number table can now also be downloaded in Excel format or retrieved via a RESTFUL-API webservice to integrate the numbering in any dataset and analysis method.

### Phylogenetic trees

This GPCRdb update features a new implementation of phylogenetic trees which can now be constructed for a larger number of receptors and bootstrapping replicas. The trees are shown as either a circular (default) or horizontal dendogram. The branches and receptor names in the tree and markers next to the receptor names can be assigned separate color-coding to map GPCR classification or functional data, such as G protein coupling preferences. The naming of receptors can be shown according to UniProt, with or without species, or the International Union of Pharmacology (29). The phylogenetic trees can be downloaded as PDF, SVG or PNG images or in Newick format for visualization in a tree viewer software. A tree can also serve as a tool to select receptors for further analysis in GPCRdb. Clicking on a node adds the receptors in the given branch to one of two selection sets for which a sequence alignment or signature (see above) can be generated on the same page. Alternatively, the receptor UniProt names can be pasted into another page in GPCRdb allowing for correlation of the receptor relationships to structural or functional properties.

### Structures, refined structures and structure models

Receptor structures, previously added biannually, are now imported monthly from the PDB and complemented by refined experimental structures in which missing or distorted regions are re-modelled and mutated residues are reverted to wildtype. Receptor models are available for all human non-olfactory receptors in inactive, intermediate and active states not yet covered by an experimental structure (23) and can optionally be downloaded without loops and termini. The GPCRdb structure table has been greatly expanded with, for example, a new section with data for signalling protein complexes, more data on the structure ligand, the endogenous ligand, and annotated auxiliary proteins. To better browse the breadth of data, the receptor name column is always shown upon horizontal scrolling and sections can be hidden if not relevant for the user. The vertical filtering of structure rows has also been improved, for example to allow filtering by any author of the structure's publication. The structure table can be downloaded in Excel format for further filtering and users can copy selected UniProt or PDB identifiers for follow-up analysis in other GPCRdb resources.

To provide an assessment of the receptor structure models in GPCRdb, we have added a new ‘Structure model statistics’ page. This page provides RMSD values of the latest model (based on another receptor template) to the first experimental structure of the given receptor and state. It also provides documentation and scripts to calculate RMSD values uniformly when comparing models from other sources. Our first publication of the receptor structures models showed a better overall accuracy than the then available other model servers and databases (23) and here we extend this comparison to a new GPCR modelling resource, RosettaGPCR (30). Whereas GPCRdb contains models of all human GPCRs in their inactive, intermediate and active state, RosettaGPCR only provides models of inactive state human GPCRs. Of all experimental structures that have been released since GPCRdb published its models (23), 37 are in the inactive state but only 21 could be used to benchmark the two model resources because RosettaGPCR’s alignment.fasta files include the target structure among its templates for the other 16 models. (GPCRdb does not model receptor and states that have an experimental structure but instead provide a refined experimental structure, see above). The 21 models, based on templates from other receptors, were compared by comparing root-mean-square deviation (RMSD) values (Supplementary Spreadsheet 1). We find that GPCRdb and RosettaGPCR performs equally well (same average RMSD values) for most receptor segments, including: 7TM (both for all atoms and backbone atoms only) and the two first intracellular loops (ICL1–2). GPCRdb has better average RMSD values (0.4 Å overall difference) for helix 8 (H8). RosettaGPCR has better average RMSD values for the three extracellular loops (ECL1–3): 1.5 Å (0,8 without FZD5 from class F for which GPCRdb has not yet aligned this loop), 0.9 Å and 0.7 Å, respectively. GPCRdb is currently working on extending our pipeline to improve the modelling of loops and termini by extending the sequence alignments (used by RosettaGPCR and several other modelling servers) to additional segments and implementing additional software.

### Sequence signature tool

The new sequence signature tool identifies residue positions that are putative functional determinants based on distinct conservation in one of two sequence alignments of receptors that share and lack the function of interest, respectively. The user provides the two receptor sets which can be selected based on own data, through the new receptor selection table (above) or by copying receptor UniProt identifiers from other GPCRdb pages, e.g. the G protein coupling page. The sequence signature is calculated as the residue positions and properties (see Materials and Methods) with the highest percent difference in conservation in the function-positive and -negative receptor sets and could therefore contribute to or counteract the given function. If a residue position has multiple residue property groups with the same percent conservation difference, the sequence signature tool reports the property group consisting of the lowest number of distinct amino acids (the most distinctive and restrictive group of residues). The user can apply a cutoff to the percent conservation distinctiveness and match the obtained sequence signature to a sequence alignment of the GPCR class to identify additional receptors that may share the function. Furthermore, the mean differences with *P*-values ≤ 0.05 for each z-scale are shown in red-green coloring to distinguish the function-negative and -positive receptor sets, respectively.

**Figure 2.**
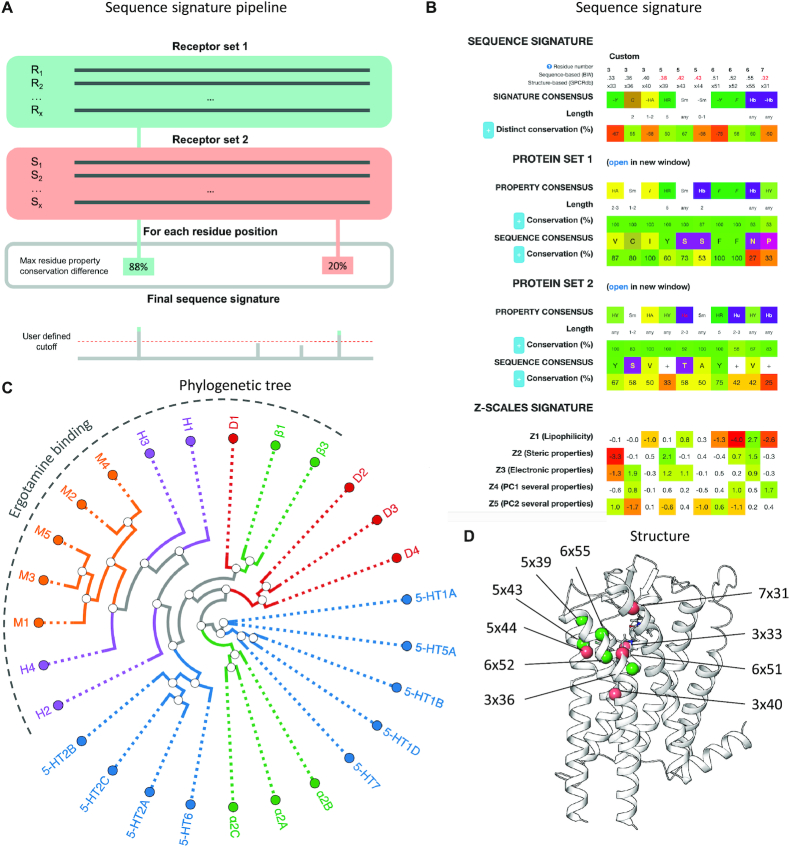
Sequence signature tool to identify residue positions that are functional determinants. (**A**) Simplified visualization of the sequence signature tool pipeline. Structure based alignments of the two receptors sets are compared to find each residue position's property with the maximum conservation difference for which the user defines a cutoff. (**B**) Results from a sequence signature analysis of aminergic receptor that bind ergotamine strongly (*K*_i_ <10 nM) or not at all (*K*_i_ >5000 nM) (31) and a percent property conservation difference cutoff set to 50%. This shows the sequence signature (top), structure-based sequence alignments of the two input sequence sets (mid) and an alternative numeric signature implementing z-scale amino acid descriptors (bottom, see Materials and Methods). Residue property details can be viewed by clicking on the (+) button. (**C**) Phylogenetic tree of the receptors used in the case study and residue positions of the amino acids interacting with Ergotamine. (**D**) Mapping of the ergotamine selectivity determinant positions (receptor sets 1 and 2 in green and orange Cα spheres, respectively) identified by the sequence signature tool onto a serotonin 5-HT_2C_ receptor structure (31).

To validate and illustrate the use of the sequence signature tool, we tested if it could reproduce a previously published study that conducted a manual sequence-structure investigation of residue determinants within aminergic receptors for binding of the agonist ergotamine (31). Ergotamine binds to α-adrenergic and the majority of serotonin and dopamine receptors with nanomolar or subnanomolar affinity but has low or no affinity for some receptor subtypes (D_1_, D_5_, 5-HT_4_ and 5-HT_1E_) in these receptor families and for all β-adrenergic, muscarinic and histaminergic receptors. Figure 2 shows the results of a sequence signature analysis of the strongest- versus non-binding receptors (*K*_i_ <10 nM and >5000 nM, respectively) and a residue distinction conservation cutoff of 50%. This reproduces the most distinctive ligand selectivity determinants from the published study: 3 × 33, 3 × 36, 5 × 43 (5.42 in sequence-based residue numbering), 6 × 51 and 6 × 52. It also discovered a distinct property, hydrogen bonding of two additional residues: 6 × 55 and 7 × 31 (7.32 in sequence-based residue numbering) and two new distinct residues in the ergotamine binding receptors: 3 × 40 (not hydrogen bond acceptor) and 5 × 44 (not small). This provides proof-of-concept for the sequence signature tool, which could be applied to identify residues determinants in any set of receptors with and without any function of interest.

## G PROTEINS, ARRESTINS and BIASED SIGNALLING (sections in development)

The published (26) parts of the G protein section of GPCRdb features G protein sequence alignments, snake-like residue topology diagrams, intersection or dissection of receptors based on their coupling to the four main G protein families and *in vitro* GPCR and G protein mutations/chimeras with effect on signalling. Further G protein data and tools, as well as wholly new arrestin and biased signalling sections, are currently in development and available in GPCRdb for testing and user feedback.

## DRUGS AND LIGANDS

### Drugs

The Drugs subsection springs from an analysis of GPCR drugs, targets and indications spanning both FDA-approved drugs and agents in clinical trials (32). It reported 475 drugs (∼34% of all approved by the FDA) for 108 GPCR drug targets, ongoing clinical trials for 321 candidate drugs and 224 (56%) non-olfactory GPCRs with untapped therapeutic potential. The associated GPCRdb resources include: (i) a table of drugs, targets & indications, (ii) mapping of drug targets onto a GPCR classification tree and (iii) drug and drug target statistics. GPCRdb aims to update these data in a coming publication and to provide a continuously updated resource (members interested in supporting annotation of these data are encouraged to contact the GPCRdb team).

### Ligands and ligand statistics

This GPCRdb update has imported the most recent version of ChEMBL (see Materials and Methods) and contains over 198 577 ligands and 393 248 dose–response (binding and functional data) values (a 37% and a 22% increase, respectively). GPCRdb enriches the ligand data by integrating physicochemical properties and their commercial availability (23). The bioactivities have herein been supplemented with information about the cell line and publication references. The current average number of ligands per receptor across the GPCR classes are: A: 567, B1: 229, C: 321, and F: 73 and the number of members with a ligand are: A: 203 (70%), B1: 14 (93%), C: 11 (50%) and F: 1 (9%).

### Ligand site mutations and mutation design tool

Since the previous GPCRdb publication (23), 4324 additional ligand site mutations, with associated effects on ligand affinity or potency, receptor surface expression or basal activity, have been annotated from literature and the current total count is 34 652. Notably, most mutations have been deposited (via a standardized Excel sheet) by the GPCR community upon or after a publication to further their dissemination and comparison to previously published experiments. This also allows authors to map mutations using GPCRdb's residue diagrams (snakeplots and helix box diagrams) or to compare mutated residues across receptors in residue tables while color-coding their effects on ligand binding or function (see (33,34)). The associated mutation design tool has been updated with the new mutagenesis data and receptor-ligand interactions from structures (see above) leading to an increase in the number of amino acids, residue positions and receptors covered by this tool.

## STRUCTURE CONSTRUCTS

The Structure Constructs section in GPCRdb integrates GPCR structure sequence from PDB, residue annotations from SIFTS (35) and an extensive manual literature annotation of mutation effects and inserts (e.g. purification tags) as well as experimental methods and reagents for GPCR structure determination (12). The automated, but not manual, data have been updated for this publication. Furthermore, a data driven construct design tool allows engineering of receptor proteins for structure determination. This is so far mainly for crystallography but also for cryo-EM studies needing terminal truncations, a hemagglutinin signal peptide to increase expression or selection of appropriate experimental conditions. Of note, the resource can also be used to assess the quality and integrity of alternative structural templates based on the protein modifications and experimental conditions and is therefore applicable for any researcher planning a structure-based modelling or functional study.

### CITE US (a citation finder)

Different GPCRdb data and tools have been described in dedicated articles as well as part of specific scientific studies for which the associated online resources serve to disseminate datasets or allow readers to repeat or update the published analyses (Table 1). Readers are asked to kindly reference this paper describing the latest GPCRdb version along with the publications of the given data and tools used. To select the appropriate references, a new section ‘Cite us’ lets users choose a given GPCRdb page to retrieve a recommended reference which is also shown at the top of the same page.

### JOIN US (annotation, development, collaboration and feedback)

To facilitate community involvement, a new section ‘Join us’ invites researchers to contribute, collaborate, share or give feedback by contacting a relevant member of our team. *Data annotation:* The GPCRdb team is looking for students and researchers that can help with annotation of literature or structure data for joint coming publications. *Database development:* The GPCRdb team is constantly looking for talented programmers either for remote collaboration, a research visit, to fill an open position or to apply jointly for a scientific project. The database and all its source code continue to be available in a fully open GitHub repository (see Availability) under an Apache 2.0 license. *Scientific collaboration:* GPCRdb can extend on publications by disseminating experimental datasets and sometimes develop tailored data or tool resources. *Social media:* To follow the latest updates and news from GPCRdb join our LinkedIn group, follow us on Twitter (@gpcrdb) or check the latest releases listed on the main page of GPCRdb. *User feedback:* We appreciate suggestions and feedback on new and existing resources.

## DATA AVAILABILITY

GPCRdb is available at https://gpcrdb.org and can also be accessed via a RESTful API, which complies with the OpenAPI specification using Swagger (code examples are available at https://docs.gpcrdb.org/web_services.html). The source code, the underlying data, and a virtual machine configuration are all available in the repositories at https://github.com/protwis/.

## Supplementary Material

gkaa1080_Supplemental_File
